# Development of a Fully Synthetic Corneal Stromal Construct via Supramolecular Hydrogel Engineering

**DOI:** 10.1002/adhm.202301392

**Published:** 2023-10-05

**Authors:** Annika F. Vrehen, Martin G. T. A. Rutten, Patricia Y. W. Dankers

**Affiliations:** ^1^ Institute for Complex Molecular Systems Department of Biomedical Engineering Laboratory of Cell and Tissue Engineering Laboratory of Chemical Biology Eindhoven University of Technology Groene Loper 7 Eindhoven 5612 AZ The Netherlands

**Keywords:** hydrogels, injectability, keratocytes, ophthalmology, stromal constructs

## Abstract

Recent advances in the field of ophthalmology show great potential in the design of bioengineered constructs to mimic the corneal stroma. Hydrogels based on synthetic supramolecular polymers, are attractive synthetic mimics of the natural highly hydrated corneal stroma. Here, a fully synthetic corneal stromal construct is developed via engineering of an injectable supramolecular hydrogel based on ureido‐pyrimidinone (UPy) moieties. The hydrogel displays a dynamic and tunable behavior, which allows for control of biochemical and mechanical cues. Two hydrogels are developed, a fully synthetic hydrogel functionalized with a bioactive cyclic arginine‐glycine‐aspartate UPy (UPy‐cRGD) additive, and a hybrid hydrogel based on UPy‐moieties mixed with collagen type I fibers. Both hydrogels supported cell encapsulation and associated cellular deposition of extracellular matrix (ECM) proteins after 21 days. Excitingly, the hydrogels support the activation of isolated primary keratocytes into stromal fibroblasts as well as the differentiation toward more quiescent corneal stromal keratocytes, demonstrated by their characteristic long dendritic protrusions and a substantially diminished cytokine secretion. Furthermore, cells survive shear stresses during an injectability test. Together, these findings highlight the development of an injectable supramolecular hydrogel as a synthetic corneal stromal microenvironment able to host primary keratocytes.

## Graphical abstract

This study demonstrates the successful development of an injectable, supramolecular hydrogel based on non‐covalently crosslinked ureido‐pyrimidinone modified oligo(ethylene glycol) monomers. The fully synthetic hydrogel supports the (re)differentiation of isolated primary keratocytes into stromal fibroblasts and differentiation toward quiescent corneal stromal keratocytes. The supramolecular artificial stromal construct presented in this work, is proposed to be an attractive tool for biomedical applications.

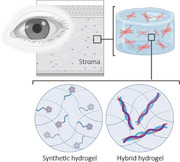

## 1 Introduction

The cornea is located at the outermost of the eye fulfilling two main functions; protecting the inner parts of the eye and allowing for vision by refracting light from the external world toward the retina. The cornea is responsible for two‐thirds of the refractive power of the eye, by maximizing light focusing on the retina and minimizing light scattering losses. These properties turn the cornea in a crucial tissue to ensure a clear vision. Disease or injury can result in a damaged cornea, and eventually cause visual regression or even blindness.^[^ ^1^, ^2^ ^]^

The corneal tissue exists of five layers; epithelial cells, the acellular Bowman's layer, stroma, the acellular Descemet's membrane and endothelial cells.^[^ ^3^ ^]^ About 90% of the cornea is composed of the corneal stroma, which predominantly affects the corneal function. The corneal stroma is a highly hydrated and organized tissue. It consists of condensed collagen type I lamellae in heterodimeric complex with collagen type V and VI, surrounded by specific proteoglycans. Collagen fibrils within successive lamellae are oriented in an orthogonal fashion. This high level of organization together with the hydration results in a specific mechanical strength and the ability to refract light.^[^ ^4^, ^5^, ^6^ ^]^ Cells in the cornea comprise 5–10% of the stromal volume and are referred to as corneal keratocytes, characterized by long dendritic protrusions. Corneal keratocytes slowly regenerate the collagen in the stroma and produce extracellular matrix (ECM) components, such as collagen type I and proteoglycans. In case of stromal defects or diseases, keratocytes can undergo metabolic activation and change into a fibroblastic phenotype, resulting in cell migration, proliferation, and upscaled collagen production.^[^ ^7^, ^8^ ^]^

The in vivo regeneration process of patients with severe stromal defects or diseases often results in uncontrollable production of disorganized fibrotic tissue due to an irreversible transformation of the keratocytes into myofibroblasts. This process often results in permanent scarring and further aggravation of the patient's visual performance.^[^ ^9^ ^]^ Treatment options for patients with severe stromal disease or injury are very limited. Many patients at risk of blindness, rely on corneal transplants from deceased human donors as the only effective available treatment. Unfortunately, there is a worldwide shortage of corneal graft tissue, with only one cornea available for seventy needed.^[^ ^10^ ^]^ In recent decades, ophthalmologists and bioengineers together are dedicated to engineer a construct, which allows to integrate into the patient's eye. Desirably, this artificial construct should trigger and support the self‐regeneration process of the native tissue, and restore the patient's vision. Due to the complexity of the stromal tissue, multiple material properties such as; transparency, biocompatibility, innervation, and the biomechanics need to be considered while engineering a stromal construct.^[^ ^11^ ^]^

Due to the viscoelastic nature and high water content, injectable hydrogels are proposed to be an attractive alternative for artificial corneas and corneal donor tissue,^[^ ^12^, ^13^ ^]^ Within the field of corneal tissue engineering there is an urgent need for readily available tissue substitutes that can precisely fill corneal defects in a minimal invasive manner.^[^ ^13^ ^]^ Very common biopolymers such as collagen, gelatin, silk, dextran, hyaluronic acid and decellularized stromal tissues are studied as potential hydrogel stromal constructs,^[^ ^8^, ^14^, ^15^, ^16^ ^]^ Although natural derived polymers are characterized by their powerful natural biochemical compositions, their stiffness is rather limited. To improve on this material limitation, hybrid hydrogels are developed. Hybrid hydrogels combine the biological power of naturally derived hydrogels with the tunability of synthetic hydrogels,^[^ ^1^, ^17^, ^18^, ^19^, ^20^, ^21^, ^22^, ^23^, ^24^, ^25^, ^26^, ^27^, ^28^, ^29^, ^30^, ^31^ ^]^ Despite the fact that these materials demonstrated great compatibility with cells and acceptable mechanical properties, they also pose a disease transmission risk, immunogenic concerns, and scalability issues. A solution to these disadvantages is to design a stromal construct via synthetic hydrogels based on supramolecular moieties, allowing for material tunability and selectivity by the incorporation of bioactive additives into the material to provide the needed biochemical cues,^[^ ^12^, ^32^ ^]^ Previously, 3D encapsulation of cells in supramolecular hydrogels was demonstrated by using a two component system containing molecules with complementary UPy groups as supramolecular building blocks.^[^ ^33^ ^]^ Via fourfold hydrogen bonding these UPy groups dimerize and form lateral stacks. In solution, these stacks assemble into fibers and transient networks are formed. Traditional synthetic hydrogels often depend on material degradability or large pore sizes to allow cellular activities such as spreading, proliferation and migration. Due to the reversibility of the non‐covalent interactions, supramolecular hydrogels are very adaptable. Within the supramolecular hydrogels, this adaptability allows for matrix remodeling and supports cellular activities, without requiring hydrogel degradation or large pore sizes.^[^ ^34^, ^35^, ^36^ ^]^

**Figure 1:**
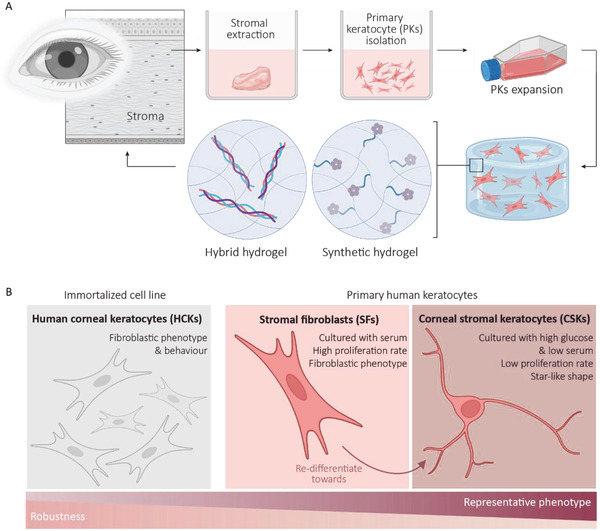
Overview of this study. A) The design of a supramolecular hydrogel as a stromal construct and the concept of the two hydrogel systems used in this study. B) Overview of the cells used in this study, the robust SV40‐immortalized human corneal keratocyte cell line and isolated human primary keratocytes which are treated toward stromal fibroblasts (SFs) or corneal stromal keratocytes (CSKs). Created with BioRender.com.

The aim of this work is to develop a hydrogel based on supramolecular chemistry that closely mimics the stromal microenvironment and induces self‐regeneration of the damaged tissue. To this end, the hydrogel design should meet some important criteria, that is, a mechanically stable environment and allowing flow of nutrients to encapsulate keratocytes. To achieve our aim, the biological and mechanical characteristics of two hydrogels are thoroughly studied and compared, namely; 1) a fully synthetic hydrogel‐based stromal construct based on UPy‐fibers and functionalized with a bioactive cyclic arginine‐glycine‐aspartate (cRGD) additive, and 2) a hybrid hydrogel‐based stromal construct composed of a combination of UPy‐fibers and natural collagen type I fibers (**Figure** **1**A). Two variations of keratocytes are used to explore the cellular biocompatibility of both hydrogel constructs, being a SV40‐immortalised human corneal keratocyte cell line (HCK) and human donor derived primary keratocytes (PKs). HCKs are more robust compared with the PKs, yet significantly less representative (Figure 1B). The HCKs are assumed to impose an irreversible phenotype of myofibroblast. Two treatments are used for the PKs. To mimic the quiescent native keratocytes, the cells are treated with culture medium supplemented with high glucose and low serum. To mimic metabolic activation of keratocytes and the transformation into fibroblasts, the PKs are treated with culture medium supplemented with high serum.

**Figure 2:**
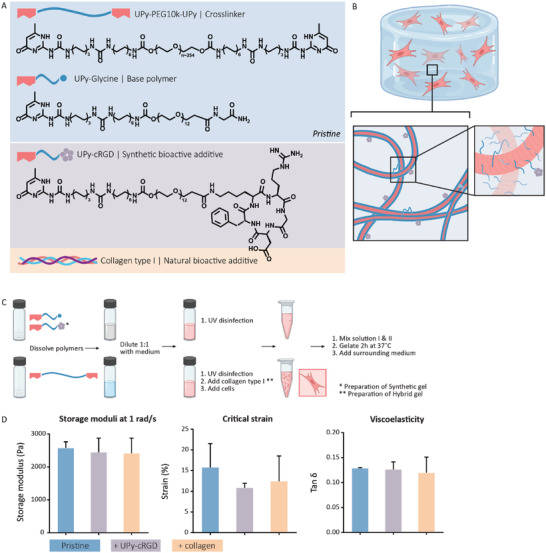
Overview of the chemical properties of the hydrogel, the formulation method of the hydrogel and rheological measurements of the three formulated hydrogels. A) The chemical structures of the hydrogelator UPy‐glycine (monofunctional molecule), the crosslinker UPy‐PEG_10K_‐UPy (bifunctional molecule), and the bioactive additive UPy‐cRGD and schematic illustration of collagen type I. B) Schematical illustration of the supramolecular network. Blue linkages between fibers indicate interfiber cross‐links formed by UPy‐PEG_10K_‐UPy. C) Hydrogel formulation method for 3D cell encapsulation. D) Mechanical properties of a pristine, synthetic and hybrid hydrogel measured after described preparation of the gels. G′, critical strain and tan(delta) values of hydrogels measured at 1 rad s^−1^ and 1% strain, *n* = 3, ±SD.

The fundament of the two supramolecular hydrogels is based on two variations of molecules with complementary UPy‐groups (**Figure** **2**A). An alkyl spacer is incorporated to shield the hydrogen bonds in water, creating a hydrophobic pocket upon assembly of UPy‐dimers into 1D stacks. Hydrogen bonding of flanking urea groups implements further stabilization of the formed stacks, and further assembly occurs by bundling of the stacks into fibers (Figure 2B),^[^ ^12^, ^33^ ^]^ The monofunctional hydrogelator, consists of a 528 Da oligo(ethylene glycol) (OEG) chain end‐capped with a functional UPy‐moiety at one end and a glycine‐amide group at the other end. Due to this molecular design of the monofunctional molecule it is expected that the glycols’ non‐fouling properties are shielded from exposure, while the glycine‐amide groups are assumed to be presented to the cells. Upon addition of a bifunctional 10 kDa poly(ethylene glycol) (PEG) chain end‐capped with two functional UPy‐moieties, interfiber cross‐links are formed.^[^ ^33^ ^]^ To introduce bioactivity into the synthetic hydrogel, a cell adhesive supramolecular additive is used based on a monofunctional molecule functionalized with an integrin‐binding cRGD ligand. Both the synthetic (+UPy‐cRGD) and the hybrid (+collagen) hydrogel are thoroughly studied in this work via various analyses of the biological and mechanical performances of both hydrogels.

## 2 Results and Discussion

### 2.1 Formulation and Mechanical Properties of Synthetic & Hybrid Hydrogels

Both the bifunctional building block and the monofunctional building block (with or without additive) are received as a powder, and dissolved separately in aqueous solutions (Figure 2C). Via fourfold hydrogen bonding between the functional UPy groups the monofunctional building blocks start to dimerize and form lateral stacks, assembling into fibers.

The solution of the bifunctional building blocks and the solution of the monofunctional building blocks are both diluted with cell culture medium to provide the cells with some nutrients during gelation. To encapsulate cells within the hydrogel, cells are supplemented to the bifunctional building block solution. Addition of the dissolved bifunctional molecules to the monofunctional fibers in solution introduces interfiber crosslinks, resulting in the formation of a transient network.

A pristine hydrogel (no additives, solely monofunctional and bifunctional molecules), a synthetic hydrogel (+UPy‐cRGD additive) and a hybrid hydrogel (+collagen) were engineered via the above mentioned preparation method (Table S1, Supporting Information, for an overview of the hydrogel compositions). Rheological measurements showed a solid‐like behavior (i.e., G′ > G″) for all the hydrogels when formulated with 2.5 wt/v% of monofunctional and bifunctional molecules in each composition (Figure S2A, Supporting Information). A complete gelation of all the hydrogels resulted in a storage modulus of ≈11 kPa for both the pristine hydrogel and the synthetic UPy‐cRGD hydrogel and a storage modulus of ≈8 kPa for the hybrid hydrogel, respectively (Figure S2B, Supporting Information). For successful cell encapsulation and to perform extended cell cultures it is necessary to embed the hydrogels in cell culture medium. This prevents desiccation of the gel and provides nutrients for the cells. Rheological measurements of pre‐formed hydrogels, incubated with medium for 24 h prior to the measurement, showed a decrease in storage moduli compared with the final storage moduli of the formation measurements. Measurements of the pre‐formed gels, which were embedded in medium, resulted in similar storage moduli of 2577 ± 185 Pa for the pristine hydrogel, 2445 ± 430 Pa for the synthetic hydrogel and 2410 ± 464 Pa for the hybrid hydrogel, respectively (Figure 2D). Furthermore, the results demonstrated similar needed critical strain and viscoelastic behavior for all the hydrogels.

### 2.2 Supramolecular Hydrogels Support Human Corneal Keratocyte Encapsulation and ECM Protein Deposition

**Figure 3:**
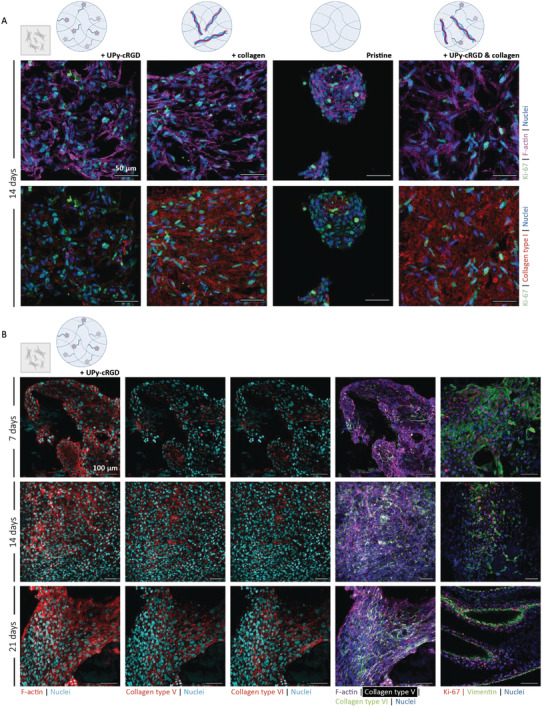
Human corneal keratocytes encapsulated and cultured for 7, 14, and 21 days within supramolecular hydrogels are able to spread, proliferate and deposit ECM proteins. A) HCKs encapsulated and cultured for 14 days in the synthetic UPy‐cRGD hydrogel and in the hybrid collagen hydrogel. The pristine hydrogel and a hybrid hydrogel functionalized with UPy‐cRGD are used as a control. Stained for ki67 (green), F‐actin (purple), collagen type I (red) and the nuclei (blue). Scale bar represents 50 µm, *n* = 2 (two hydrogels per staining). B) HCKs encapsulated and cultured in the synthetic hydrogel for 7, 14, and 21 days. Stained for F‐actin, collagen type V, collagen type VI, Ki‐67 and vimentin. Scale bar represents 100 µm, *n* = 2 (two hydrogels per staining).

A SV40‐immortalised human corneal keratocyte cell line (HCK) was used as a simplified cell model to explore the possibilities of the supramolecular hydrogels,^[^ ^37^, ^38^ ^]^ At first, the HCKs were encapsulated within the synthetic and hybrid hydrogel and cultured for 14 days. As a control the cells were also encapsulated within the pristine hydrogel (without cell adhesive additives) and within a hybrid hydrogel functionalized with UPy‐cRGD (**Figure** **3**A). After a 14‐day culture, HCKs encapsulated within the synthetic and the hybrid hydrogel showed the expected elongated morphology of the HCKs. Proliferation marker Ki‐67 marked proliferating HCKs in both hydrogels, suggesting the cells are still proliferating after 14 days of encapsulation. Furthermore, collagen type I protein deposition was supported by the synthetic hydrogel and the results indicate a homogenous mixed‐in collagen type I for the hybrid hydrogel. Based on these results, no differences in biological performance between the synthetic and hybrid hydrogel were observed. A round‐shaped cell morphology was observed for the HCKs encapsulated within the pristine hydrogel. In addition, the cells clustered together, forming cell clumps instead of a homogenous distribution of spreaded cells throughout the gel. These observations demonstrated the need to incorporate essential biochemical cues within the hydrogels, to allow the keratocytes to maintain their healthy morphology. The hybrid hydrogel functionalized with UPy‐cRGD did not improve the biological performances of the hydrogel. These results demonstrated that the incorporation of both additives did not led to a reinforcement of the hydrogels biological performance.

To further analyze the biological performance of the synthetic hydrogel, the HCKs were encapsulated and cultured for 7, 14 and 21 days to perform some additional immunohistochemical analyses (Figure 3B). During culture, an increase in cellular deposition of collagen type V and collagen type VI was observed between day 7 and day 21. At all the timepoints, some cells expressed proliferation marker Ki‐67, indicating a continuous proliferation of the cells.

### 2.3 Primary Keratocyte Encapsulation and Steering of Phenotype

**Figure 4:**
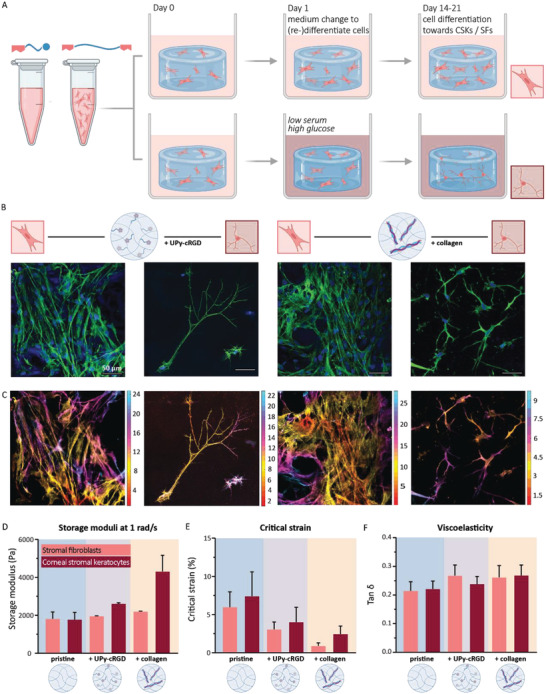
Human PKs encapsulated and cultured for 21 days in supramolecular hydrogels, *n* = 3. A) Experimental set up and timeline. B) Fluorescent confocal images of PKs treated with serum toward SFs and PKs treated with high glucose and low serum toward CSKs, when encapsulated in the synthetic and hybrid hydrogel. Stained for vimentin (green) and nuclei (blue). Scale bar represents 50 µm, *n* = 2 (two hydrogels per condition). See Figure S5A, Supporting Information, for cell images containing a higher cell density of the CSKs. C) Multicolored images show the cell heights. Scale is in µm. See Figure S5B, Supporting Information, for the height indication of the associated nuclei. D) Storage moduli at 1 rad s^−1^, *n* = 2, ±SD. E) Critical strain, *n* = 2, ±SD. F) Viscoelastic behavior, *n* = 2, ±SD. Created with Biorender.com.

Human primary keratocytes (PKs) are isolated from human donor corneal stroma and expanded in vitro. The PKs are successfully encapsulated into the synthetic and hybrid system (day 0). Subsequently, after 1 day of culture (day 1) the PKs are treated in two manners; 1) with serum toward stromal fibroblasts (SFs) or 2) with low serum and high glucose toward quiescent corneal stromal keratocytes (CSKs).^[^ ^39^ ^]^ The cellular encapsulation was prolonged to 21 days to allow successful (re‐)differentiation of the PKs (**Figure** **4**A). PKs encapsulated within the synthetic and hybrid hydrogel, treated with high serum, attain a fibroblastic phenotype and exhibit a higher proliferation rate (Figure 4B). A substantial different cellular visualization is observed for the encapsulated PKs treated with high glucose and low serum. The results demonstrated hydrogels loaded with fewer cells compared with the high serum treated PKs (Figure S4A, Supporting Information). The keratocytes show long dendritic protrusions that allow them to sense the surrounding matrix and other cells. Especially, the cells encapsulated within the synthetic hydrogel possess long protrusions of ≈ 300–350 µm (Figure 4B). Besides the visual morphological difference between the different treated PKs, the results demonstrate a difference in tubulin β3 expression as well. The expression of this neural differentiation marker is slightly increased for the PKs treated with high glucose and low serum (Figure S3A,B, Supporting Information). Multicolored images show the differences in cell height. As expected for cells cultured in a 3D environment, these results demonstrated the ability of the cells to spread and migrate toward all directions (Figure 4C). Without the addition of bioactive additives the PKs were not able to adhere to the hydrogel, only a few cell clumps consisting of small round‐shaped cells were observed on day 7 (Figure S3C, Supporting Information). At culture day 21, rheological measurements were performed on hydrogels loaded with encapsulated cells. Due to the variation in bioactive additive as well as the provided supplements in the cell culture medium, these measurements were performed to explore any signs of differences in cellular rearrangement of the hydrogel‐networks. However, the rheological measurements demonstrate that the storage moduli, critical strain and viscosity of the pristine, synthetic and hybrid hydrogel are all within a similar range to each other (Figure 4D–F). Similar results are observed for the complete frequency sweep, strain sweep and stress‐relaxation curve (Figure S6, Supporting Information). The rheological measurements were also repeated with PKs derived from another donor, again the results were all in similar range to each other and comparable to the first dataset (Figure S7, Supporting Information).

### 2.4 PKs Slightly Experience Mechanotransduction when Encapsulated in Supramolecular Hydrogels

**Figure 5:**
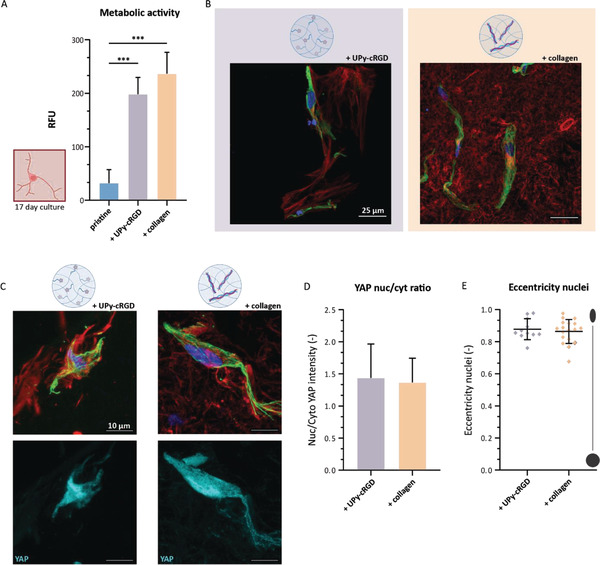
Encapsulated PKs treated toward CSKs sensed similar mechanotransduction when encapsulated in the synthetic or hybrid hydrogel and cultured for 17 days. A) Metabolic activity measured after 17‐day culture showing almost no activity for the cells in the pristine hydrogel. *n* = 3, *** *p* < 0.0005, one way Anova and Tukey's multiple comparisons test, ±SD. B) Immunohistochemical staining of F‐actin (green), collagen type I (red) and nuclei (blue). Scale bar represents 25 µm. C) Immunohistochemical staining of F‐actin (green), collagen type I (red), and nuclei (blue), top row. And YAP signal (cyan), bottom row. Scale bar represents 10 µm. D) YAP nucleus/cytoplasm ratio, for both gels ≈1.4, ±SD, *n* = 3. E) Nucleus eccentricity, for both hydrogels between 0.8 and 1.0, ±SD, *n* = 3. Additional cell data is provided in Figure S12, Supporting Information.

PKs encapsulated within the synthetic and hybrid hydrogel, treated with high glucose and low serum, experience a surrounding gel‐matrix with a stiffness of ≈2–4 kPa (Figure S8C,D, Supporting Information). Mechanotransduction allows cells to sense and adapt to external forces by cytoskeleton remodeling or activating specific genetic programs.^[^ ^40^ ^]^ To explore the interaction between the cells and the material in more detail, an analysis of yes‐associated‐protein (YAP) is implemented in this study. YAP is a mechanosensitive transcriptional regulator, which is on a mechanical level regulated by mechanical cues such as ECM rigidity, strain or adhesive area.^[^ ^41^ ^]^ Pathways involving YAP translocation to the nucleus, allow the cells to perceive ECM mechanics and to spread.^[^ ^42^ ^]^ At first, the metabolic activity of the CSKs was measured via a resazurin assay. These results demonstrated metabolic active CSKs on day 17 (**Figure** **5**A). Furthermore, the results of the synthetic hydrogel demonstrated the ability of both hydrogels to support the elongated keratocyte morphology as well as the cellular deposition of collagen type I. A homogenous distribution of the mixed‐in collagen type I and a similar elongated cell morphology were observed for the keratocytes cultured within the hybrid hydrogel (Figure 5B). A custom‐made cell profiler pipeline was used to quantify the intensity of YAP expression inside the cytoplasm and inside the nuclei (Figure S8A,B, Supporting Information). The ratio's between nuclear YAP intensity and YAP intensity within the cytoplasm of the CSKs encapsulated within the synthetic and hybrid hydrogel are both above 1 (Figure 5C,D). In addition, the nuclei eccentricity value of the CSKs encapsulated in both hydrogel systems is also within the same range of ≈0.8–1.0, suggesting that the nuclei are oval‐shaped instead of round‐shaped (Figure 5E). Both the higher nuclear YAP intensity as well as the oval‐shaped nuclei suggest that the cells do experience mechanotransduction. Yet, between the synthetic and hybrid hydrogel there is no difference in mechanotransduction sensed by the encapsulated PKs.

### 2.5 (Re‐)Differentiation of Primary Keratocytes into Corneal Stromal Keratocytes

**Figure 6:**
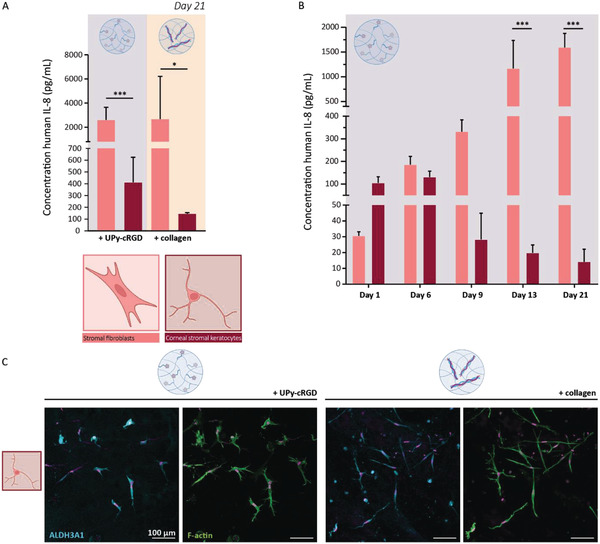
PKs (re)differentiated toward SFs and CSKs when encapsulated and cultured in the synthetic and the hybrid hydrogel. A) Interleukin 8 (IL‐8) secretion by PKs encapsulated in the synthetic and hybrid hydrogel at culture day 21. Controls of this experiment can be found in Figure S10A,B, Supporting Information. *n* = 3, *p* < 0.0005 *** and *p* < 0.05 *, unpaired *t*‐test, ±SD. B) IL‐8 secretion of PKs cultured in the synthetic UPy‐cRGD hydrogel at day 1, 6, 9, 13, and 21. Control of this experiment can be found in Figure S10C, Supporting Information. *n* = 3, *p* < 0.0005 ***, unpaired *t*‐test, ±SD. C) Immunofluorescent staining of ALDH3A1 (cyan), F‐actin (green), and nuclei (magenta). Scale bar represents 100 µm.

During the 21‐day cell culture of encapsulated cells, small amounts of cell culture medium were stored to perform an enzyme‐linked‐immunosorbent assay (ELISA). From literature it is known that SFs secrete several cytokines, that is, interleukin‐8 (IL‐8), interleuking‐15 (IL‐15) and RANTES/CCL5. All of these cytokines are pro‐inflammatory molecules, and important for neutrophil recruitment and T‐cell activation. In contrast, CSKs hardly secrete these cytokines. To this end, an ELISA to measure IL‐8 secretion was used to analyze the cellular (re‐)differentiation during 3D cell culture of 21 days within the synthetic and hybrid hydrogel.^[^ ^43^ ^]^ PKs encapsulated within the synthetic and hybrid hydrogel, treated with high serum toward SFs, secreted >2600 pg mL^−1^ IL‐8. While, PKs encapsulated within the synthetic and hybrid hydrogel, treated with low serum and high glucose toward CSKs, secreted <415 pg mL^−1^ IL‐8. These results demonstrated a distinct difference in cellular processes and behavior between the PKs treated toward SFs and the PKs treated toward CSKs (**Figure** **6**A). Cellular IL‐8 secretion was not affected by the different bioactive additives within the two hydrogels, for both the synthetic and the hybrid hydrogel the results demonstrated a substantial decrease in IL‐8 secretion for PKs treated toward CSKs. The analysis of IL‐8 secretion at various time points during the cell culture of 21 days, demonstrated a progressive decrease of IL‐8 secretion by the PKs treated toward CSKs (Figure 6B). In contrast, the PKs treated toward SFs, demonstrated a progressive increase of cellular IL‐8 secretion during culture. This behavior was expected for the SFs, since these cells are highly proliferative and therefore the cell number increases during culture time and simultaneously the concentration of cytokines secreted will increase. The cellular secretion of IL‐8 at various time points in the cell culture demonstrated a change in the cellular behavior of the PKs treated toward CSKs. The cellular secretion of IL‐8 by the PKs treated toward CSK is significantly lower compared with the secreted IL‐8 by the PKs treated toward SFs. This differences could be caused by the difference in cell proliferation and associated cell numbers, however dividing the IL‐8 concentration by the cell concentration demonstrated a rather limited influence of cell proliferation (Figure S4B, Supporting Information). The experiment was repeated with PKs of another donor (Figure S10D,E, Supporting Information) and the same trend in IL‐8 secretion was observed for these cells.

Healthy human keratocytes do also express crystallin proteins that reduce light scattering and thereby enhance the optical performance of the stromal tissue. One of these proteins is the enzyme aldehyde dehydrogenase 3 Family member A 1 (ALDH3A1), the most common corneal crystallin in mammals,^[^ ^44^, ^45^ ^]^ To this end, ALDH3A1 is assumed to be a specific marker for keratocytes. Here, immunohistochemical staining showed the expression of ALDH3A1 in PKs treated toward CSKs (Figure 6C), suggesting that the encapsulated PKs successfully differentiated into CSKs.

### 2.6 Cells Survived Shear Stresses during Injectability Test

**Figure 7:**
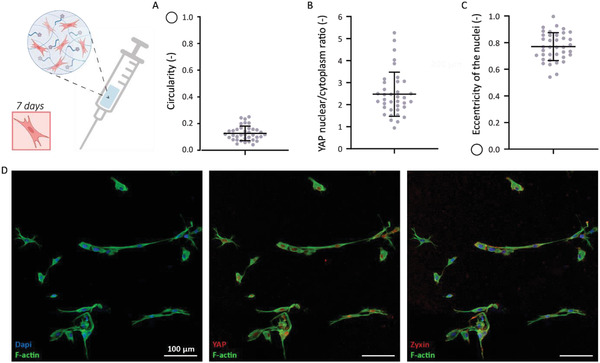
Exploring the injectability of the hydrogels resulted in properly formed gels containing spreaded PKs, *n* = 3. A) Cell morphology expressed in circularity, toward 1 indicates a round‐shaped cell and toward 0 indicates a spreaded cell, ±SD. B) YAP nuclei/cytoplasm ratio, ≈2.5, ±SD. C) Nuclei eccentricity of ≈0.75, ±SD. D) After 7 days Immunofluorescent staining was performed, the cells were stained for vimentin (green), nuclei (blue), and YAP / zyxin (red). Scale bar represents 100 µm. See Figure S9, Supporting Information, for the independent channel of YAP.

Above mentioned results are all obtained by using standard pipette tips and precise mixing of the polymer solutions per well. To eventually turn the supramolecular artificial stromal construct into an attractive tool for biomedical applications, we here focused on the clinical applicability of the hydrogel. for this purpose, the injectability of the stromal construct was explored. A needle with a diameter of 0.16 mm was used to study the injectability (Figure S11A,B, Supporting Information). 2 days post the encapsulation of cells in the injected synthetic hydrogel, the results showed cell survival (Figure S11C, Supporting Information). After 7 days of culture, immunohistochemical staining indicated that the cells survived and slowly were able to attain their healthy morphology (**Figure** **7**A,D). Expression of YAP is clearly observed inside the nuclei (Figure S11, Supporting Information), and analysis of the YAP expression showed a mean ratio of ≈2.5 between nuclear YAP intensity and YAP intensity within the cytoplasm (Figure 7B). A nuclear eccentricity of ≈0.75 indicates the nuclei are oval‐shaped instead of round‐shaped (Figure 7C). Together these results demonstrated that the cells do experience mechanotransduction. It is proposed that, this increase in YAP nuclear/cytoplasm ratio could be triggered by the experienced shear stress during the injection through the needle. However, the cells were able to survive and spread, prolonged cell culture is necessary to increase knowledge about the long term impact of the injection procedure on cellular behavior.

## 3 Conclusion

This work demonstrates the successful engineering of a fully synthetic corneal stromal construct via supramolecular hydrogel engineering. Here, we compared the differences in rheological properties, cell encapsulation, cell culture and cell differentiation between a fully synthetic hydrogel and a hybrid hydrogel, and demonstrated no substantial differences between both hydrogels. This is surprising, due to the substantial differences in bioactivity between the presence of small bioactive UPy‐cRGD additives within the synthetic hydrogel, and the presence of full length collagen type I fibers within the hybrid hydrogel. The fully synthetic hydrogel showed to be an attractive candidate as a fully synthetic stromal ECM mimicking material suitable to encapsulate PKs. Encapsulation and culture of representative PKs, which were treated toward CSKs, showed long dendritic protrusions, expressed the keratocyte specific marker ALDH3A1 and demonstrated a lower cell concentration indicating a slower proliferation rate. Moreover, 3D cultured PKs treated toward CSKs hardly secreted any cytokines. Taken together, these cellular characteristics demonstrated the (re)differentiation of PKs encapsulated and cultured within the fully synthetic hydrogel. Toward clinical use of the synthetic hydrogel, injectability properties were studied. PKs survived the shear stresses during the injection trial, demonstrating the ability of using the synthetic stromal hydrogel construct as an attractive minimal invasive tool to fill tissue defects or to introduce corneal cells and initiate self‐regeneration. In conclusion, the synthetic stroma has shown to be a functional and mechanically stable microenvironment, allowing inflow of nutrients to successfully encapsulate native keratocytes.

## 4 Experimental Section

### Preparation of Hydrogels

Both the bifunctional building blocks as well as the monofunctional building blocks were received as powders. Bifunctional molecules (UPy‐PEG10K‐UPy) were dissolved at 70 °C in an neutral PBS solution for 1.5 h. Monofunctional molecules (UPy‐Glycine or UPy‐Glycine + UPy‐cRGD) were dissolved at 70 °C in an alkaline PBS solution (containing 160 mm NaOH) for 20 min. After the powders were completely dissolved, the solutions were cooled down to room temperature. A specific volume of HCl solution (2 m) was added to the monofunctional solution to reach neutral pH. For hybrid hydrogel preparation collagen type I (Gibco, Collagen I bovine, A1064401) was added to the bifunctional molecule solution, 20 µL was added per 100 µL complete gel (0.1 wt/v%). Upon addition of the bifunctional molecule solution to the monofunctional molecule solutions gelation was initiated, resulting in hydrogel formation. Incubation of the hydrogels at 37 °C allowed a proper gelation of the, and after 1 h the hydrogels were embedded in PBS to prevent dehydration.

### Rheological Measurements

A discovery hybrid rheometer (DHR‐3, TA Instruments) was used for all the rheological measurements of supramolecular solutions (formations measurements in Supporting Information) and pre‐formed hydrogels.

### Measuring Pre‐Formed Hydrogels without Cells

Hydrogels were made via above mentioned protocol inside a polystyrene 96 well F‐bottom cell culture microplate (Greiner bio‐one, 655 180). After formation, the gels were left 24 h incubated in PBS at 37 °C. For measurement, gels were transferred onto the peltier plate and a flat stainless‐steel plate‐plate geometry (diameter = 8 mm) was used. The geometry was slowly lowered, to prevent sample damage, until the sample completely filled the geometry, resulting in a gap height of 625–1050 µm. Low viscosity oil (47 V 100, RHODORSIL) was applied to seal the gap around the hydrogel to minimize evaporation or drying during the measurements which were performed on 37 °C.

### Measuring Pre‐Formed Hydrogels with Cells

Hydrogels were made via the below mentioned cell encapsulation protocol, inside a polystyrene 96 well F‐bottom cell culture microplate (Greiner bio‐one, 655 180). Prior to the measurement, during cell culture, the gels were stored embedded in medium inside a cell culture incubator at 37 °C, 2% O2 and 5% CO2 for 17 or 21 days, respectively. On the day of the measurement the medium on top of the gels was removed and the gels were washed 1× with PBS. Subsequently the gels were transferred to the rheometer and a flat stainless‐steel plate‐plate geometry (diameter = 8 mm) was used. The geometry was slowly lowered, to prevent sample damage, until the sample completely filled the geometry, resulting in a gap height of 625 – 1050 µm. Low viscosity oil (47 V 100, RHODORSIL) was applied to seal the gap around the hydrogel to minimize evaporation or drying during the measurements which were performed on 37 °C.

### Measuring Rheological Characteristics

Strain sweep measurements (1–1000% strain, 1 rad s^−1^) were performed to determine the linear viscoelastic region of hydrogels. Frequency sweeps were measured with frequencies ranging from 100 to 0.1 rad s^−1^, at a constant strain of 1%. Time sweeps were measured at a constant frequency and a constant strain of 1 rad s^−1^ and 1%. Stress relaxation experiments were performed by applying a strain of 7.5%, and measuring the generated stress for 1000 s. The data were normalized using the stress generated after 1 s.

### Human Corneal Keratocyte Cell Culture

Human corneal keratocyte (HCK) cell lines were obtained as a kind gift from Dr. Zorn‐Kruppa (University Medical Center Hamburg). The HCK cell line was derived from the human corneal stroma and immortalized through SV‐40 transfection, and has been previously demonstrated to mimic both the phenotype and the response to growth factor stimulations of their primary precursors,^[^ ^37^, ^38^ ^]^ HCK cells were cultured in Dulbecco's Modified Eagle's Medium (DMEM, Sigma‐Aldrich) supplemented with 5% FBS (Biochrom), and 1% penicillin/streptomycin at 37 °C, 21% O_2_ and 5% CO_2_ until ±80% confluency was reached. Due to activation of the cells by the FBS supplementation, the HCK cells can be considered activated keratocytes, resembling a fibroblastic phenotype. Cells were passaged twice a week, and cells between passages 5 and 10 were used for the experiments described.

### Primary Keratocyte Cell Culture

Primary human keratocytes were isolated from leftover human corneoscleral transplant material from Descemet Membrane Endothelial Keratoplasty surgery, which were obtained from the Cornea Department of the ETB‐BISLIFE Multi‐Tissue Center (Beverwijk, the Netherlands). The keratocytes were cultured in expansion medium (1:1 mixture of Dulbecco's modified Eagle's medium/F‐12 supplemented with GlutaMAX (DMEM/F12 (Ham) + GlutaMAXTM, 10565‐018; Gibco), 5% Fetal Bovine Serum (FBS, Biochrom AG), 1% penicillin/streptomycin (P/S, Biochrom AG), and 1 mm L‐ascorbic acid 2‐phosphate sesquimagnesium salt hydrate (Vitamin C, Sigma A8960)) at 37 °C, 21% O_2_ and 5% CO_2_ until ±80% confluency. Since the medium contained FBS, keratocytes were considered to be activated matrix‐producing cells, referred to as stromal fibroblasts (SFs). To initiate cell (re‐)differentiation toward corneal stromal keratocytes, another medium composition was used: differentiation medium (Dulbecco's modified Eagle's medium supplemented with GlutaMAX (GlutaMAXTM, 11880‐028; Gibco), 1% penicillin/streptomycin (P/S, Biochrom AG), and 1 mm L‐ascorbic acid 2‐phosphate sesquimagnesium salt hydrate (Vitamin C, Sigma A8960), 1× ITS (Sigma, I3146), 2 mg mL^−1^ D‐glucose (Invitrogen, 15 023 021), 2.5 mg mL^−1^ D‐mannitol (Fluka, 63 560)).^[^ ^21^ ^]^ Cells were cultured with medium changes evert 3 days, keratocytes from multiple donors were used up to passage# 3. TrypLE Express Enzyme (1×), no phenol red (12 604 013, Gibco) was used to detach the cells from the culture flask and use them for experiments.

### Cell Encapsulation in Hydrogels

Both the bifunctional as well as the monofunctional building blocks were received as powders. The bifunctional molecules (UPy‐PEG_10K_‐UPy) were dissolved at 70 °C in an neutral PBS solution for 1.5 h. Monofunctional molecules (UPy‐Glycine or UPy‐Glycine + UPy‐cRGD) were dissolved at 70 °C in an alkaline PBS solution (containing 160 mm NaOH) for 20 min. After completely dissolving the powders, the solutions were cooled down to room temperature. A specific volume of HCl solution (2 m) was added to solution of monofunctional molecules, to reach a neutral pH. Cell culture medium was added to both solutions 1:1, to provide the cells already with some nutrients during the gelation process later on in the procedure. Afterward, both solutions are transferred from a glass vial to a sterile Eppendorf tube, from this step onward a safety cabinet is used to guarantee a sterile work environment. The solutions were disinfected by exposing them to UV‐light for 20 min. Subsequently, the cells were prepared and counted, the following cell concentrations were used during the experiments:

### HCKs: 100 cells µL^−1^

Primary keratocytes treated toward SFs: 100 cells µL^−1^

Primary keratocytes treated toward CSKs: 200 cells µL^−1^

The cells needed for encapsulation are suspended in the correct amount of medium and this cell suspension was added to the solution of bifunctional molecules. Due to the addition of the cells suspension, the bifunctional molecules were diluted. To correct for this extra dilution step, the initial concentration of the bifunctional molecules was slightly higher (0.78 wt/v% instead of 0.52 wt/v%). For every experiment the bifunctional molecules were diluted with 1/3 of cell suspension, resulting in a final 0.52 wt/v% of bifunctional molecules. All the gels were prepared in wells of a non‐adhesive 96‐well plate (Fisher Scientific, Nunclon Sphera‐Treated, U‐Shaped‐Bottom plate 15 227 905).

### Synthetic Hydrogel

First, 40 µL monofunctional molecules in solution were added to a well. Meanwhile, the cells were added to the solution of bifunctional molecules and mixed thoroughly. Second, 40 µL of bifunctional molecules / cell mixture were added to the 40 µL solution of monofunctional molecules inside the well. The molecules were mixed by carefully pipetting up and down (at least 3× per well), any air bubbles were removed by using a needle.

### Pristine Hydrogel

First, 40 µL monofunctional molecules in solution were added to a well. Instead of adding the cells, only medium was added to the solution of bifunctional molecules and mixed thoroughly. Second, 40 µL of bifunctional molecules / medium mixture were added to the 40 µL solution of monofunctional molecules inside the well. The molecules were mixed by carefully pipetting up and down (at least 3× per well), any air bubbles were removed by using a needle.

### Hybrid Hydrogel

For hybrid hydrogel preparation collagen type I (Gibco, Collagen I bovine, A1064401) was added to the solution of the bifunctional molecules, 20 µL was added per 100 µL complete gel (0.1 Wt/v%). Initially, the bifunctional molecules were dissolved at a higher wt/v% compared with the bifunctional molecules used for the synthetic or pristine hydrogel, to correct for this extra dilution step with the collagen. At first, 40 µL solution of monofunctional molecules was added to a well. The cells and the collagen were added to the solution of bifunctional molecules and mixed thoroughly. Second, 40 µL of bifunctional molecules / cell / collagen mixture were added to the 40 µL solution of monofunctional molecules inside the well. The molecules were mixed by carefully pipetting up and down (at least 3× per well), any air bubbles were removed by using a needle.

All the hydrogels were placed in the incubator at 37 °C, 21% O2 and 5% CO2 for 1 h to allow for proper gelation. After 1 h, medium was carefully added to the wells to embed the gels, and the hydrogels with encapsulated cells were placed back in the incubator at 37 °C, 21% O2 and 5% CO2. After 1 day the cell culture medium was refreshed and to treat the PKs toward CSKs, the expansion medium was replaced by the differentiation medium.

### Cell Staining and Imaging

Before immunohistochemical stainings were carried out, the hydrogels were washed 3× with PBS (5 min per wash). All cells encapsulated within the hydrogels were fixated for 20 min at room temperature using 3.7% paraformaldehyde (formalin 37%, 104 033.1000, Merck). After washing with PBS, samples were permeabilized for 15 min with 0.5% Triton X‐100 in PBS. Followed by adding a blocking solution of 10% donkey or goat serum in 0.05% Triton X‐100 in PBS for 30 min. Next, the cells were incubated with the primary antibodies diluted in 2% donkey serum in 0.05% Triton X‐100 in PBS overnight at 4 °C. Thereafter, the cells were washed thoroughly with 0.05% Triton X‐100 in PBS, including wash waiting steps of 5–10 min. Next, the cells were incubated with the secondary antibodies and phalloidin at room temperature for 2 h. Finally, the cells were stained with DAPI at a dilution of 1:250 for 10 min and washed thoroughly with PBS (including was waiting steps of 5–10 min). During imaging, complete/intact hydrogels were place on a thin coverslip (24 × 69 mm, VWR 631–1575) immerged in mowiol 4–88 (Sigma Aldrich, 81 381) and imaged using Leica TCS SP8 X inverted confocal microscope (Leica Microsystems) using HC PL APO CS2 objectives (20×/0.75, 40×/0.95). Images were processed in ImageJ to create a max‐projection image of the original z‐stack. See **Table** **1** for the primary and secondary antibodies used within this study.

**Table 1:** Overview of the used dyes, primary‐, and secondary antibodies.

Antibody / dye	Company / reference #	Dilution
4’,6‐diamidino‐2‐phenylindole dihydrochloride (DAPI)	Sigma‐Aldrich, D9542	1:250
Phalloidin 488	Sigma‐Aldrich	1:300
Phalloidin 555	Sigma‐Aldrich	1:300
Anti‐Collagen type I	C2456, Sigma	1:250
Anti‐Collagen type V	1350‐01, Southem Biotech	1:250
Anti‐Collagen type VI	70R‐CR009x, Fitzgerald	1:250
Anti‐Vimentin	Ab20346, Abcam	1:300
Anti‐Ki‐67	Rb1510‐P0, Thermo Scientific	1:200
Anti‐ tubulin β3	801202, BioLegend	1:100
Anti‐YAP1	Ab52771, Abcam	1:100
Anti‐ALDH3A1	Ab76976, Abcam	1:100
Anti‐ZYX	HPA004835, Atlas antibodies	1:100
*Secondary antibodies*		
Anti‐mouse IgG1 (goat) 555	A21127, Molecular Probes	1:250
Anti‐mouse IgG1 (goat) 488	A21121, Molecular Probes	1:250
Anti‐goat IgG (donkey) 488	A11055, Molecular Probes	1:250
Anti‐rabbit IgG (donkey) 555	A31572, Molecular Probes	1:250
Anti‐rabbit IgG (donkey) 488	A21206, Molecular Probes	1:250
Anti‐rabbit IgG (donkey) 647	711‐605‐152 Jackson	1:250
Anti‐rabbit IgG (goat) 647	A21244, Molecular Probes	1:250
Anti‐rabbit IgG (goat) 555	A211428, Molecular Probes	1:250
Anti‐mouse IgG2a (goat) 555	A21137, Molecular Probes	1:250
Anti‐mouse IgM (goat) 488	A21042, Molecular Probes	1:250
Anti‐mouse IgM (goat) 555	A21426, Molecular Probes	1:250

All stainings were performed with a combination of solely donkey or goat secondary antibodies. Donkey serum was used for a combination of solely donkey‐based secondary antibodies, goat serum was used for a combination of solely goat‐based secondary antibodies.

### Metabolic Activity

Prior to the YAP‐analysis a resazurin assay was performed to check the metabolic activity. Primary keratocytes (PKs) of passage #2 were encapsulated with a cell density of 30.000 cells per 100 µL hydrogel. The PKs were cultured for 17 days using high glucose and low serum differentiation medium, which was refreshed every 2–3 days. On day 17, at first a resazurin assay was performed, afterward the hydrogels were thoroughly washed and 1 gel of each condition was used for rheological measurements, the other ones were fixated for immunohistochemical staining. First, the gels were washed with PBS. Afterward the gels were transferred to a new 24 well plate (each gel in a separate well). To each well 500 µL of 44 µm resazurin in medium was added and incubated at 37 °C for 3 h. Subsequently, 2 × 200 µL of the incubated resazurin/medium was collected and transferred to a black 96‐well plate to measure fluorescence (excitation: 530/25 and emission: 590/35) with the plate reader (Synergy HTX, BioTek).

### Enzyme‐Linked Immunosorbent Assay

On various days during the 3D cell culture, medium was refreshed and all medium surrounding the sample was collected in separate small Eppendorf tubes and stored at −80 °C. All ELISA experiments were executed with *n* = 3, and all the standards as well as the samples were ran in duplicate. The following IL‐8 concentrations were used for the standard curve of IL‐8: 1000, 500, 250, 125, 62.5, 31.3, and 15.6 pg mL^−1^.

Uncoated NuncMaxiSorp ELISA plates (BioLegend, 423 501) were used, these plates were coated with capture antibody diluted in 1× coating buffer 1 day prior to running the ELISA (overnight incubation at 4 °C). See **Table** **2** for all the compositions of the used reagents. On the day of the experiment, all the samples, the plates and the ELISA MAX Deluxe Set components (BioLegend) were brought to room temperature. All the washing steps were executed in a similar manner, namely: 4 times with at least 300 µL wash buffer (0.05% Tween‐20 in PBS) per well and residual buffer was blotted by firmly tapping the plate upside down on absorbent paper. After incubation with the capture antibody, the plate was washed and blocked with 1× assay diluent A at room temperature, for 1 h with shaking (500 rpm) to minimize non‐specific binding and reduce background. During this incubation, the samples for the standard curve were prepared. After blocking, the wells were washed and incubated for 2 h with shaking (500 rpm) with 100 µL of the samples and standards at room temperature. All samples were diluted 4× in 1× assay diluent (25 µL sample, 75 µL 1× assay diluent). Next, the wells were washed and 100 µL of detection antibody solution was added to each well, and incubated at room temperature for 1 h with shaking (500 rpm). The wells were washed again and incubated with 100 µL of diluted Avidin‐HRP solution at room temperature for 30 min with shaking (500 rpm). Subsequently, the wells were washed 5× thoroughly with wash buffer, and soaked in buffer for 1 min for each wash to minimize background. Then, 100 µL of substrate solution C was added and incubated in the dark for 15 min. During this step, positive wells turned blue in color. To stop the reaction, 100 µL Stop Solution for TMB Substrate (BioLegend, 4 230 021) was added to each well. All the positive wells turned from blue to yellow during this step. After adding the stop solution to the wells, the plate was softly tapped to the table a few times to proper mix the solutions and immediately the absorbance at 450 nm was measured with the plate reader (Synergy HTX, BioTek). In addition the absorbance at 570 nm was measured, this absorbance could be subtracted from the 450 nm absorbance. The standard curve for each ELISA was plotted in excel and polynomial curve‐fitting software with order 3 or 4 was used to determine the best fit. Using this formula together with the measured absorbance value, resulted in the calculation of the amount of cytokine secretion.

**Table 2:** Reagents used to perform an ELISA with 2× complete 96‐well plate.

Reagents used for 2× 96 well plates *Material*	*Diluted with*
4 mL Coating Buffer A (5×)	16 mL of Deionized Water
100 µL of Capture Antibody (200×)	20 mL of 1× Coating Buffer
12 mL of Assay Diluent A (5×)	48 mL of PBS
100 µL of Detection Antibody (200×)	20 mL of 1× Assay Diluent A
20 µL of Avidin‐HRP (1000×)	20 mL of 1× Assay Diluent A
Substrate Solution C	‐

### Used ELSIA Set

ELISA MAX Deluxe Set Human IL‐8 (BioLegend, 431 504)

### Injection of Hydrogel

Initially this protocol was similar to the preparation of hydrogels protocol. The proper dissolved and neutral solutions of M‐type molecules and the B‐type molecules were placed on ice in separate Eppendorf tubes. A clean Eppendorf tube, small syringe and a 30G needle were placed on ice as well. At first, both molecule solutions were mixed 1:1 together in the clean and pre‐cooled Eppendorf tube and afterward the tube was placed back on ice. Subsequently, the needle and syringe were used to collect the molecule mixture and to inject the mixture in an empty petri dish/well (Figure S11, Supporting Information). The injected hydrogel was placed in the incubator at 37 °C to form properly. After 1 h, surrounding medium was added and it was observed that the gel stayed intact, suggesting a proper formation. The following cell concentrations were used; 200 cells µL^−1^ gel and 400 cells µL^−1^ gel. Live staining was carried out according to the manufacturer's protocol (Thermo Fisher Scientific) using calcein‐AM to stain for live cells on day 2 of the culture. Afterward on day 7, immunohistochemical staining was carried out as described above and cell image analysis was carried out as described below.

### Cell Image Analysis

All image analysis were analyzed from maximum‐intensity z‐projections of confocal image stacks. Cell count (Figure S4, Supporting Information) was analyzed from images obtained after nuclei staining. To this end, ImageJ software was used to determine the number of nuclei per image. To determine the localization of YAP, z‐projections of images were obtained from channels corresponding to the DAPI (nuclei), phalloidin (F‐actin) and anti‐YAP antibody staining (YAP). Afterward CellProfiler (cell image analysis software) was used to design a pipeline which determined the nuclear areas and cytoplasmic areas. The mean intensity was determined in those respective areas and the ratio between the concentration present in nuclear and cytoplasmic regions of cell was then used as a measure of YAP nuclear translocation. The same pipeline was also used to determine the nuclear eccentricity and the cell circularity (4π × (cell area / perimeter of the cell2)).

### 3D Height Indication Cell Images

A custom MatLab script was written to pair a specific height within the obtained z‐stack image to a specific color. Both image stack obtained from channels corresponding to the DAPI (nuclei) and phalloidin (F‐actin) were used to create these 3D height indication cell images. LAS X Life Science Microscope Software was used to calculate the original µm distance in the z‐direction from the original lif file.

### Statistical Analysis

To test the significant differences in metabolic activity, a Shapiro‐Wilk test was performed to prove a normal distribution of the data, *n* = 3. An one way Anova and Tukey's multiple comparisons test was performed with Graphpad Prism. Differences were considered significant for *p*‐values < 0.05. Values are presented as an average ± standard deviation. *** *p* < 0.0005 (0.0004). The YAP and eccentricity analyses were not significant.

### Elisa Assay

To test the significant differences of the results of the Elisa assay, a Shapiro‐Wilk test was performed to prove a normal distribution of the data, *n* = 3. An unpaired *t*‐test was performed with Graphpad Prism. Differences were considered significant for *p*‐values < 0.05. Values are presented as an average ± standard deviation. *** *p* < 0.0005 (0.0003), * *p* < 0.05 (0.0378).

## Conflict of Interest

The authors declare no conflict of interest.

## Supporting information

Supporting Information

## Data Availability

The data that support the findings of this study are available from the corresponding author upon reasonable request.
