# Supplementary material for: Development of a Fully Synthetic Corneal Stromal Construct via Supramolecular Hydrogel Engineering
Source: Adv Healthc Mater. 2023 Oct 5;12(32):2301392. doi: 10.1002/adhm.202301392 (PMC11468521; doi:10.1002/adhm.202301392)
Supplement: Supplementary file 1 — Supporting Information [file ADHM-12-2301392-s001.pdf]

ADVANCED HEALTHCARE MATERIALS

Supporting Information

for *Adv. Healthcare Mater.*, DOI 10.1002/adhm.202301392

Development of a Fully Synthetic Corneal Stromal Construct via Supramolecular Hydrogel Engineering

*Annika F. Vrehen, Martin G. T. A. Rutten and Patricia Y. W. Dankers**

Supporting Information

Development of a Fully Synthetic Corneal Stromal Construct via Supramolecular Hydrogel Engineering

*Annika F. Vrehen, Martin G. T. A. Rutten, Patricia Y. W. Dankers**

Table S1. Overview of the three studied hydrogel compositions, namely; pristine hydrogel, synthetic hydrogel and hybrid hydrogel.

**Hydrogel compositions**					
**Pristine hydrogel (no additives)**					
Polymer / additive	*Ratio*	*µmol*	*mM*	*Wt/v %*	
UPy-PEG$_{10K}$-UPy	1	0.56	0.23	0.26	
UPy-Glycinamide	80	45.0	18.75	2.24	
**Synthetic hydrogel (+UPy-cRGD**					
Polymer / additive	*Ratio*	*µmol*	*mM*	*Wt/v %*	
UPy-PEG$_{10K}$-UPy	1	0.56	0.23	0.26	
UPy-Glycinamide	67.2	37.8	15.75	1.88	
UPy-cRGD	12.8	7.2	3.0	0.52	
**Hybrid hydrogel (+collagen)**					
Polymer / additive	*Ratio*	*µmol*	*mM*	*Wt/v %*	*Concentration (mg/ml)*
UPy-PEG$_{10K}$-UPy	1	0.56	0.23	0.26	
UPy-Glycinamide	80	45.0	18.75	2.24	
Collagen				0.1	1

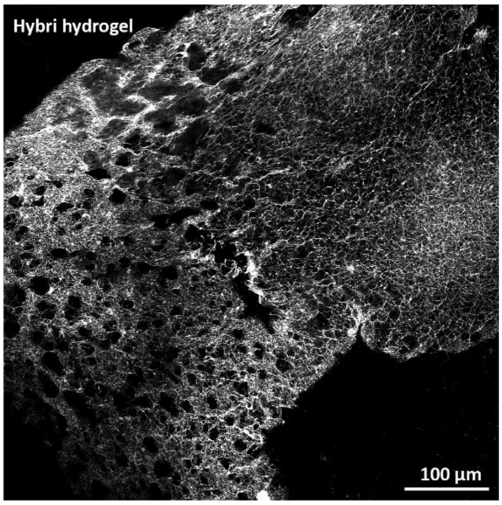

Figure S1. Visualization of collagen type I within the hybrid hydrogel. CNA35 is used to stain the collagen.

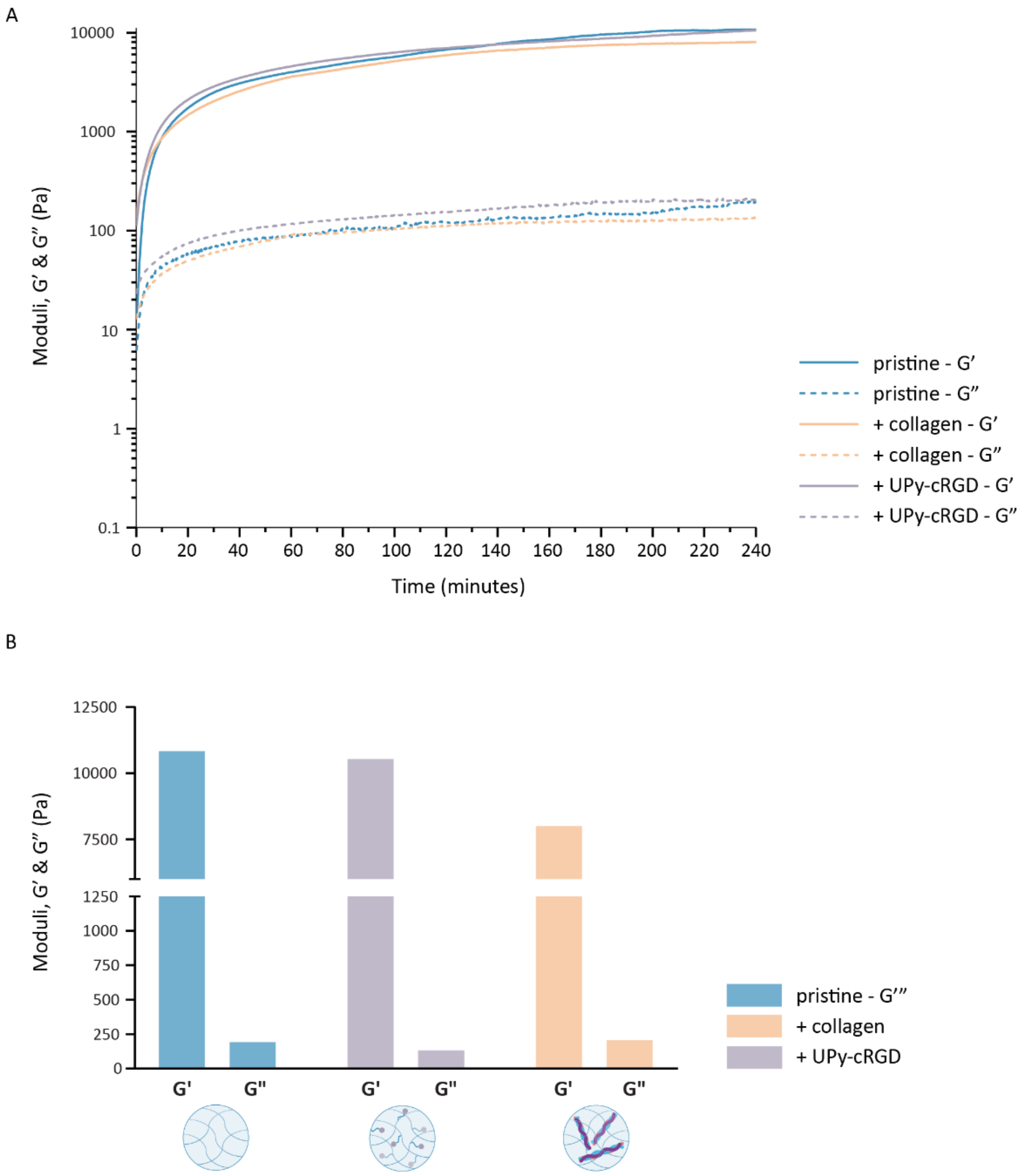

Figure S1. Rheological properties of the pristine, synthetic and hybrid hydrogel after mixing the hydrogelators on top of the rheometer plate. Hydrogel formation and measurements at 37°C, n=1. A) Time-sweep measured over a time span of 4 hours, B) the storage (G’) and loss (G”) moduli after 4 hour formation.

Measuring formation of hydrogels

B-type and M-type solutions were prepared as mentioned above and provided in two separate vials on ice. A 20 mm aluminum cone-plate geometry (2.007°) was used at a truncation gap of 56 µm. Samples were loaded by mixing the B-type and M-type solutions in an Eppendorf tube and pipetting the mixture directly on the Peltier plate, heated to 37°C. A solvent trap was use to minimize sample drying and/or evaporation.

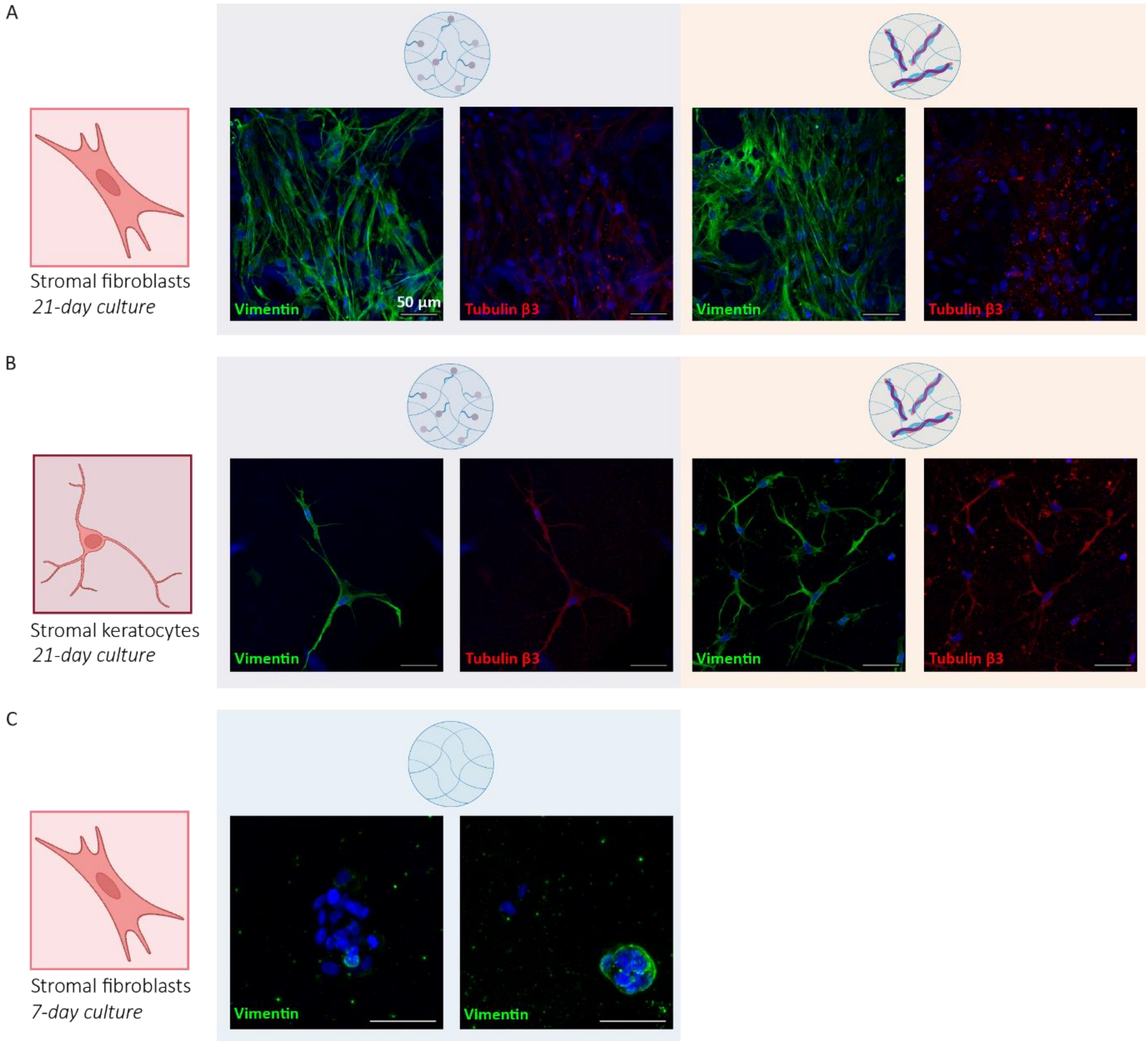

Figure S3. The synthetic and hybrid hydrogel are compatible with primary keratocytes (PKs). Fluorescent confocal images of cells cultured for 21 days in hydrogels. Stained for vimentin (green), tubulin β3 (red) and nucleus (blue), scale bars are 50 µm, n=2. A) Primary keratocytes treated with serum towards stromal fibroblasts. B) Primary keratocytes treated with low serum ad high glucose towards corneal stromal keratocytes. C) Primary keratocytes encapsulated and cultured in a pristine hydrogel for 7 days, treated with serum towards stromal fibroblast.

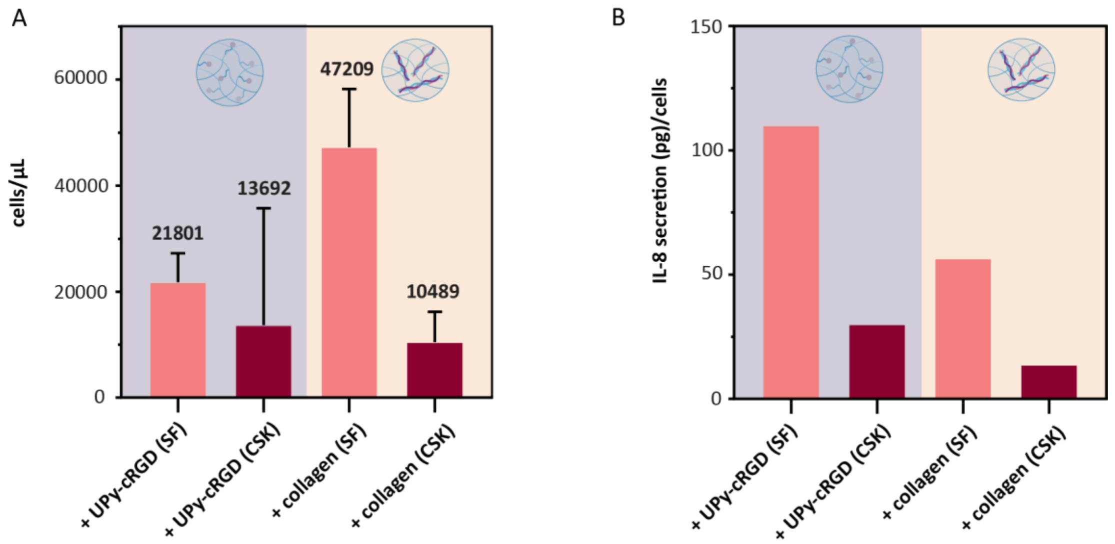

Figure S4. Concentration of cells within a hydrogel and the impact of cell proliferation on cellular IL-8 secretion. A) Quantification of the number of cells within a hydrogel. n=2, six images used per hydrogel, counted cells were corrected for the volume of the z-stack. Initially started with 100 cells/µL for the gels loaded with PKs treated towards SFs and 200 cells/µL for the gels loaded with PKs towards CSKs. B) Concentration of IL-8 secreted by the cells (pg/mL) divided by the concentration of cells (cells/mL)

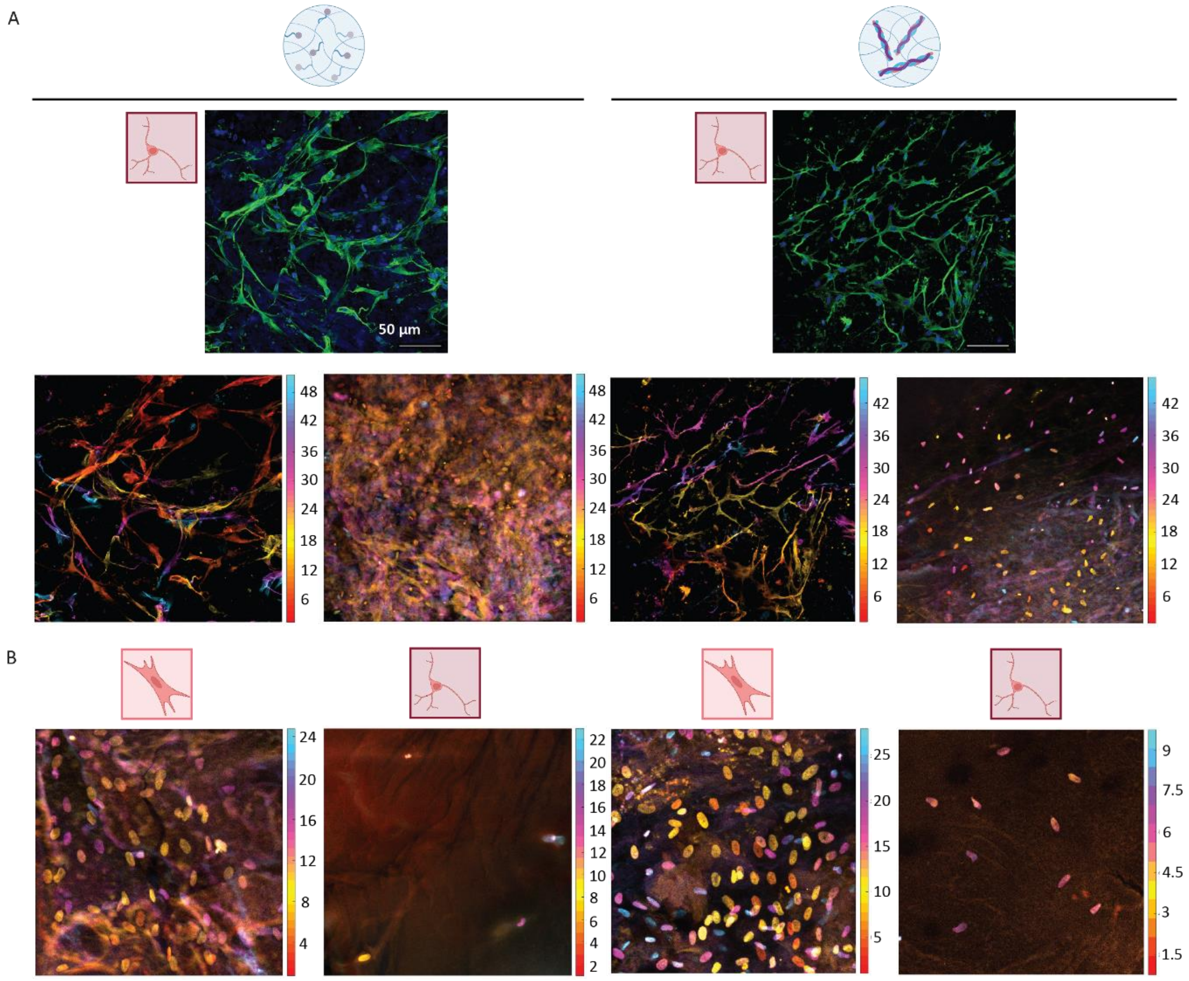

Figure S5. Additional images belonging to figure 4 in the main text. A) Higher cell density images of the primary keratocytes treated with low serum towards CSKs. The cell images in the main text indicate the clear morphological differences between the PKs treated towards SFs and CSKs. Here, images are shown of the CSKs in a higher cell density, still clear differences in cell morphology are observed when compared with the cell images of the SKs. The cells are more isolated and possess less cell-cell contacts compared with the SFs. Bottom row represents multicolored images which demonstrate the cell height in the acquired Z-stack of both the vimentin and the nuclei channel. Color scale in µm. B) Multicolored images which demonstrate the height of the cellular nuclei, the images obtained from the vimentin channel are visible in figure 4C of the main text. Color scale in µm.

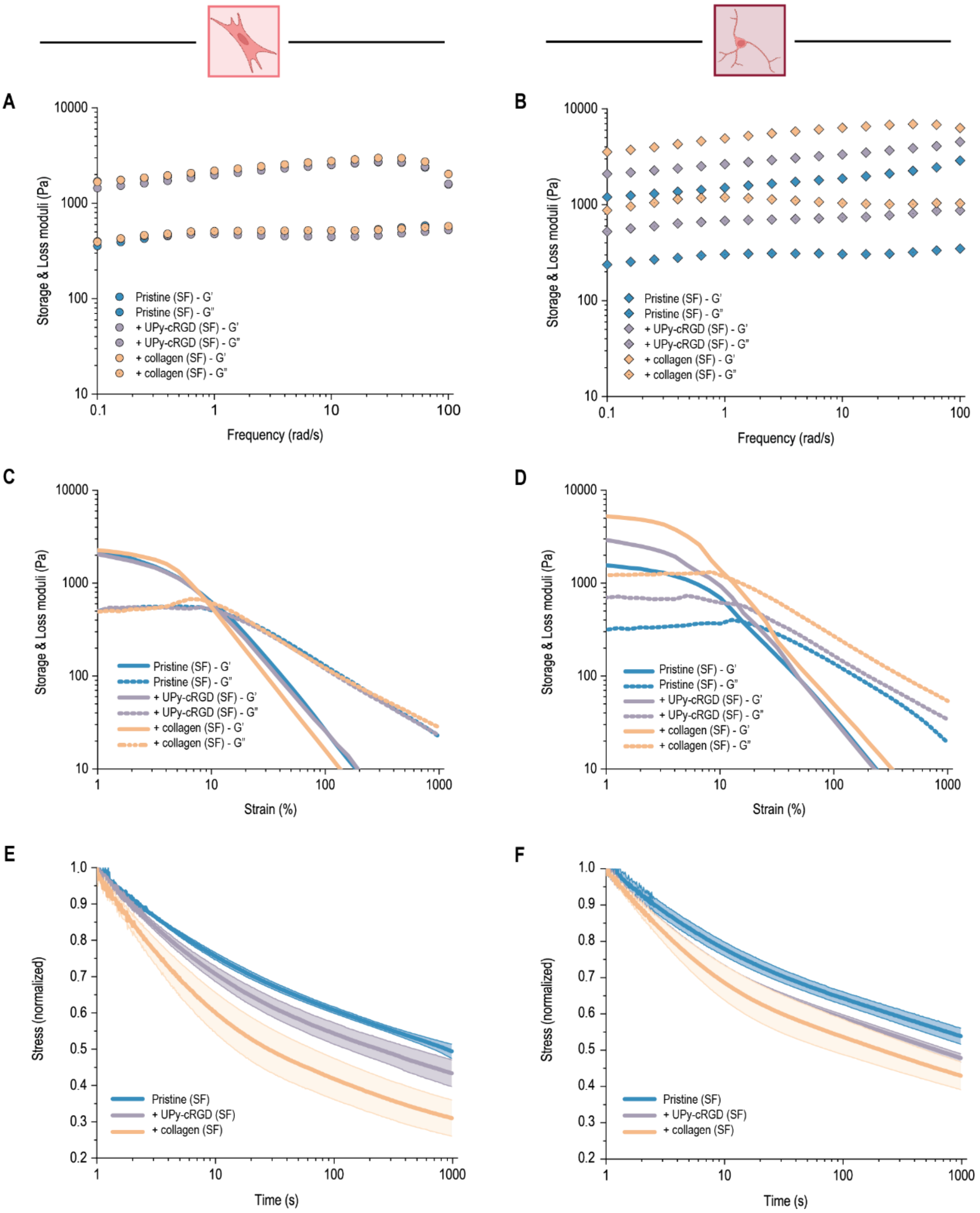

Figure S6. The mechanical properties of the pristine, synthetic and hybrid hydrogel with primary keratocytes encapsulated and cultured for 21 days. Measured at 37°C, n=2. Showing A) the frequency sweep comparing the hydrogels for the PKs treated as stromal fibroblasts, B) the frequency sweep comparing the hydrogels for the PKs treated as stromal keratocytes, C) the strain-sweep comparing the hydrogels for the PKs treated as stromal fibroblasts, D) the strain-sweep comparing the hydrogels for the PKs treated as stromal keratocytes, E) stress-relaxation at 1% strain, measured over a time span of 1000 s, comparing the hydrogels for the PKs treated as stromal fibroblasts, F) the stress-relaxation at 1% strain, measured over a time span of 1000 s, comparing the hydrogels for the PKs treated as stromal keratocytes

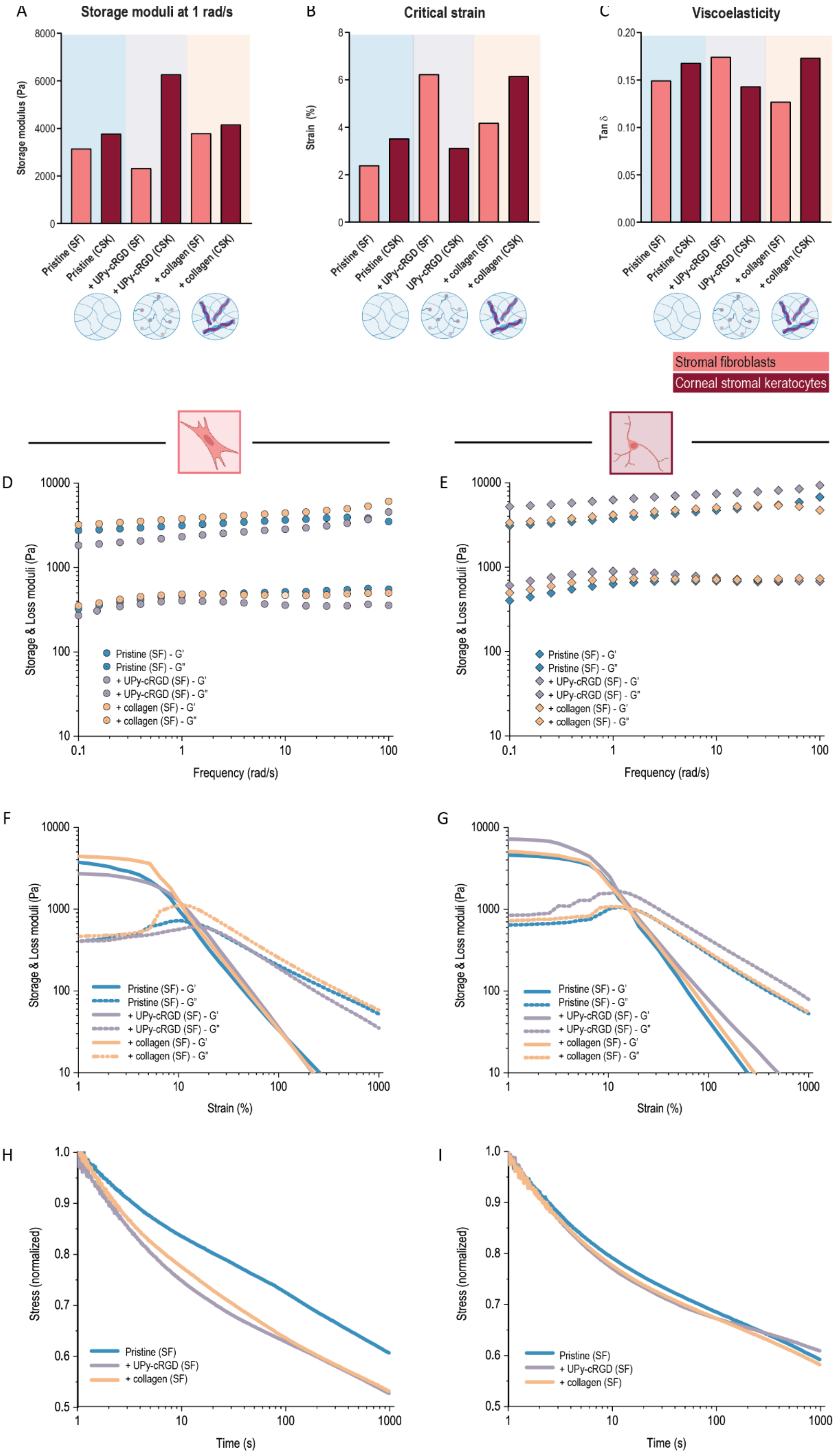
A
Storage moduli at 1 rad/s
Storage modulus (Pa)
B
Critical strain
Strain (%)
C
Viscoelasticity
Tan δ
Pristine (SF)
Pristine (CSK)
+ UPy-cRGD (SF)
+ UPy-cRGD (CSK)
+ collagen (SF)
+ collagen (CSK)
Stromal fibroblasts
Corneal stromal keratocytes
D
E
Storage & Loss moduli (Pa)
Frequency (rad/s)
Pristine (SF) - G'
Pristine (SF) - G"
+ UPy-cRGD (SF) - G'
+ UPy-cRGD (SF) - G"
+ collagen (SF) - G'
+ collagen (SF) - G"
F
G
Strain (%)
H
I
Stress (normalized)
Time (s)
Pristine (SF)
+ UPy-cRGD (SF)
+ collagen (SF)

.

Figure S7. The mechanical properties of the pristine, synthetic and hybrid hydrogel with primary keratocytes encapsulated and cultured for 21 days. Measured at 37°C, n=2. Showing A) the storage moduli at 1 rad/s, B) the critical strain, C) the viscoelasticity, D) the frequency sweep comparing the hydrogels for the PKs treated as stromal fibroblasts, E) the frequency sweep comparing the hydrogels for the PKs treated as stromal keratocytes, F) the strain-sweep comparing the hydrogels for the PKs treated as stromal fibroblasts, G) the strain-sweep comparing the hydrogels for the PKs treated as stromal keratocytes, H) the stress-relaxation at 1% strain, measured over a time span of 1000 s, comparing the hydrogels for the PKs treated as stromal fibroblasts, I) the stress-relaxation at 1% strain, measured over a time span of 1000 s, comparing the hydrogels for the PKs treated as stromal keratocytes.

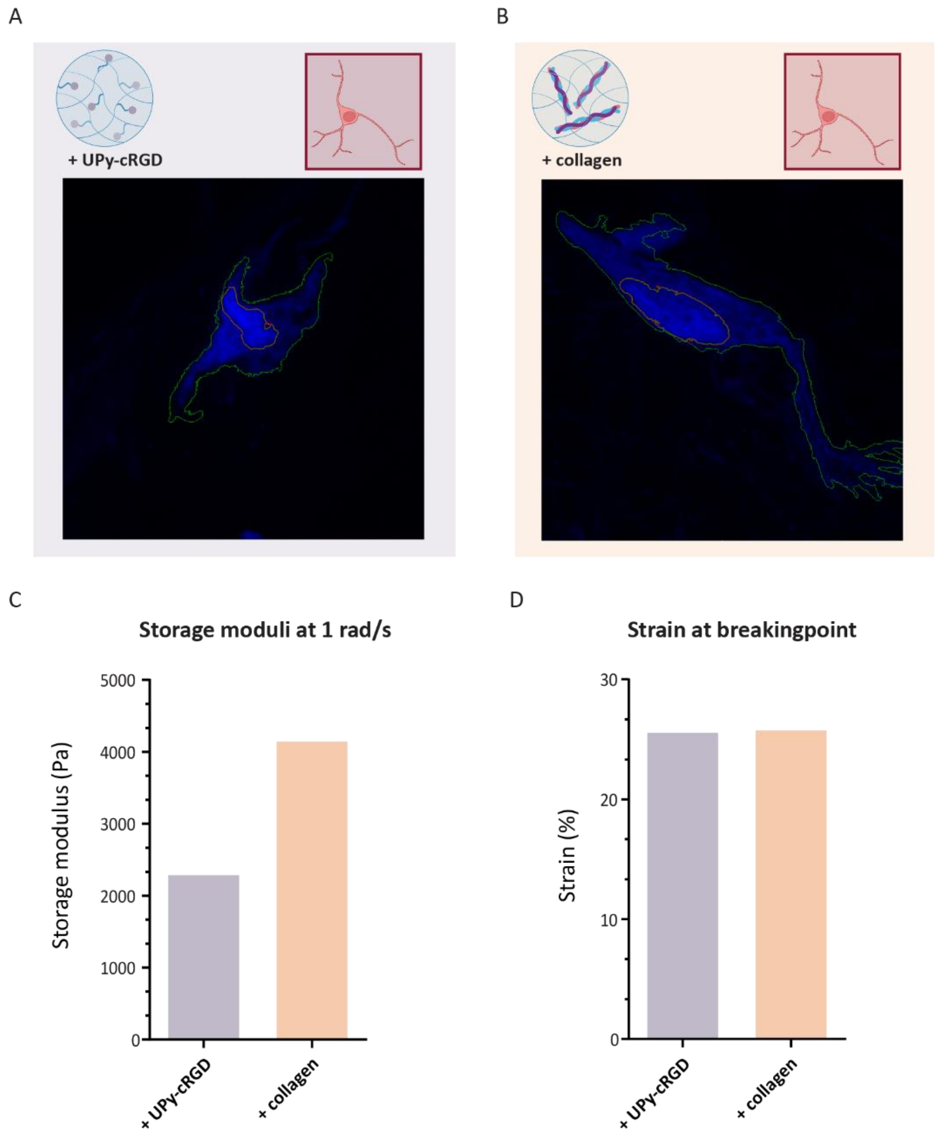

Figure S8. Additional data supporting Figure 5. A) Cellprofiler™ was used to determine the intensity of YAP signal in the cytoplasm (green) and the YAP intensity in the nucleus (red). YAP signal in blue, cell encapsulated and cultured for 17 days in the synthetic hydrogel. B) Cellprofiler™ was used to determine the intensity of YAP signal in the cytoplasm (green) and the YAP intensity in the nucleus (red). YAP signal in blue, cell encapsulated and cultured for 17 days in the hybrid hydrogel. C,D) Mechanical characteristics of the hydrogels after a 17-day culture period, n=1. Matching the data represented in Figure 4 and SI Figure 6.

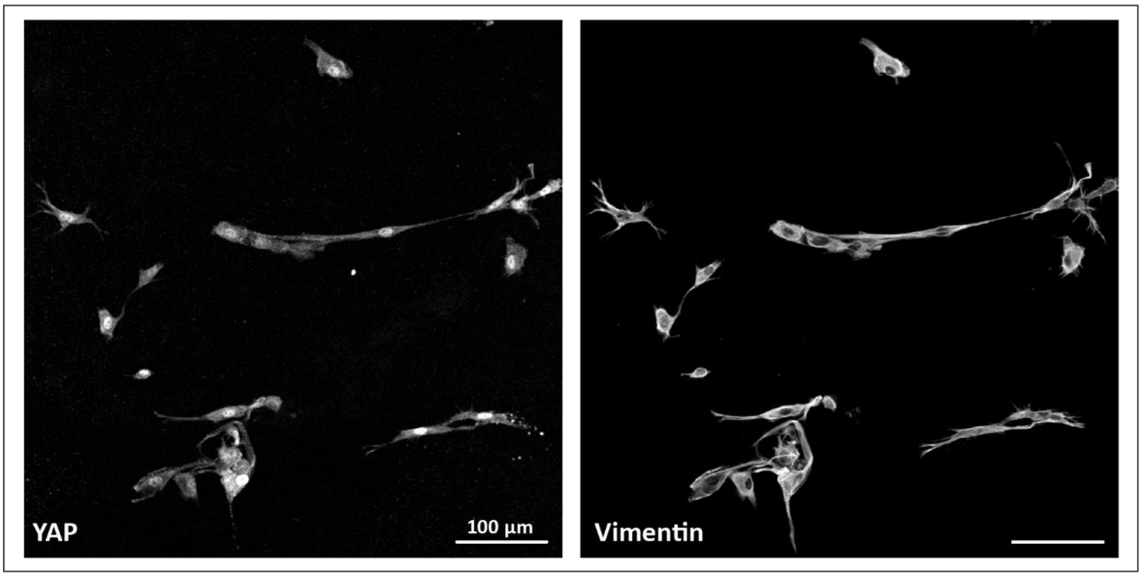

Figure S9. Independent channels of YAP and vimentin. Additional data of figure 7 in the main text.

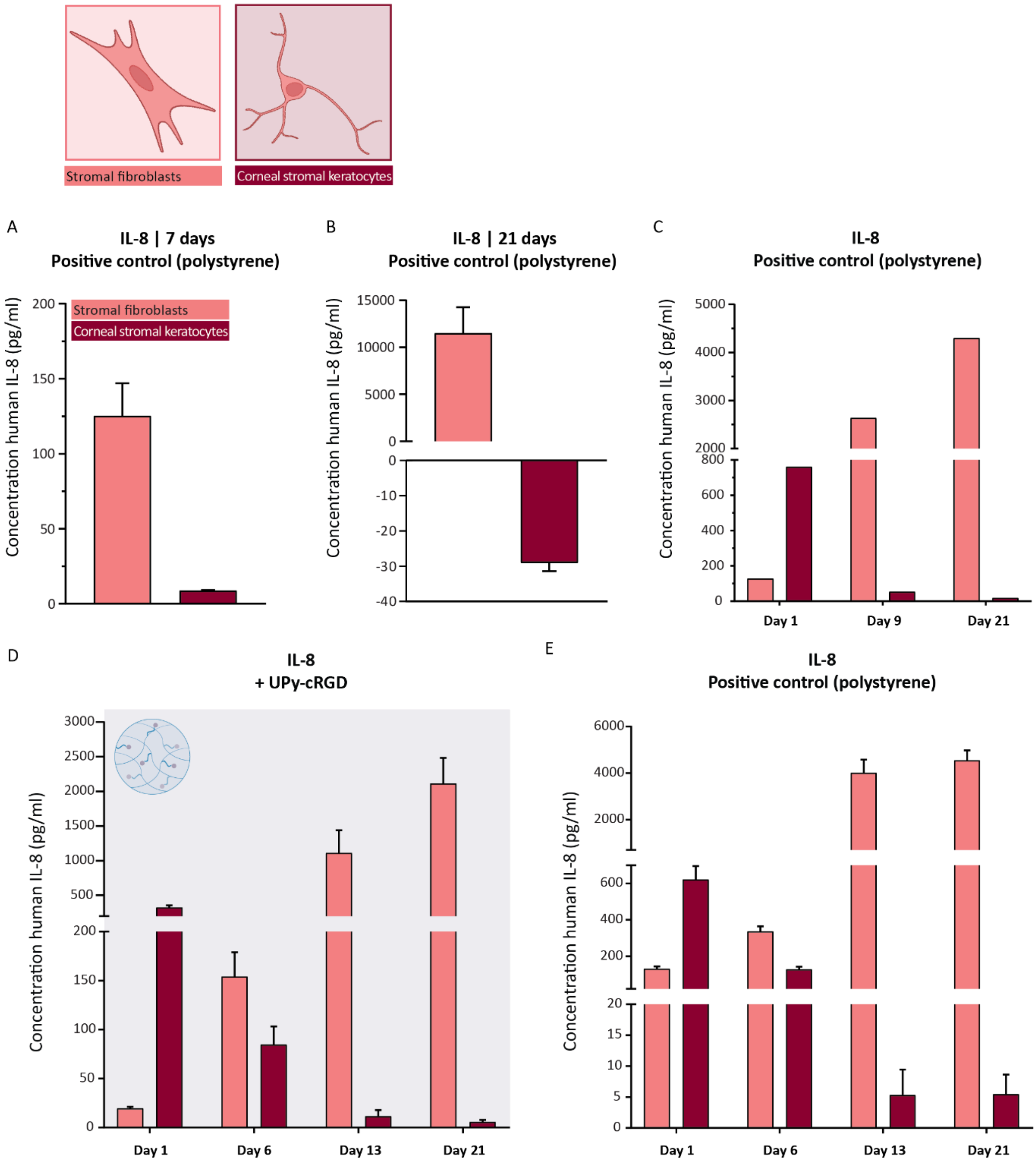

Figure S10. Additional data of IL-8 secretion by PKs. A) Positive control of data shown in figure 6A. IL-8 secretion on day 7 of PKs cultured on normal culture treated polystyrene, n=3. B) Positive control of data shown in figure 6A. IL-8 secretion on day 21 of PKs cultured on normal culture treated polystyrene, n=3. C) Positive control of data shown in figure 6B. IL-8 secretion on day 1, 9 and 21 of PKs cultured on normal culture treated polystyrene, n=1. D). IL-8 secretion of PKs cultured in the synthetic hydrogel at day 1, 9 and 21 (another donor), n=3. E) Positive control of data shown in figure D, n=3.

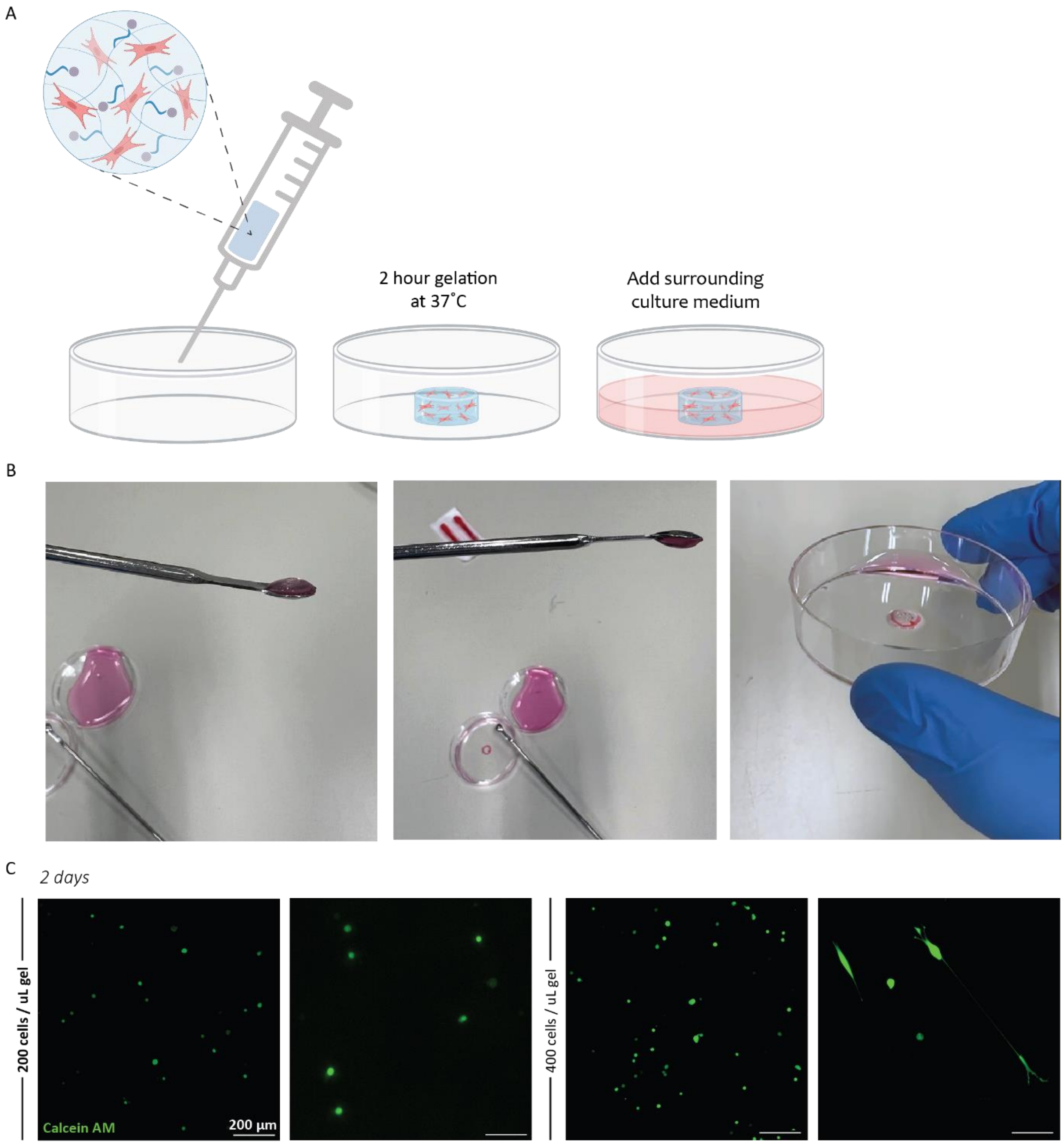

Figure S11. Successfully exploring the injectability of the hydrogel system. A) The separate hydrogel solutions are prepared as described previously (main text Figure 2B). Subsequently the two solutions are mixed and put on ice and sucked into the syringe. Thereafter the solution is injected via a 30G needle into the Petri dish, incubated at 37°C for 2 hours and embedded in medium. B) The surrounding medium is removed again and the hydrogel remains in the dish. Some pictures to show how the gel can be handled by using small spoons. C) Two days after the injection live staining was performed using calcein AM, only livings cells are able to convert calcein AM to a green-fluorescent calcein after acetoxymethyl ester hydrolysis by intracellular esterases. Scale bar represents 200 µm.

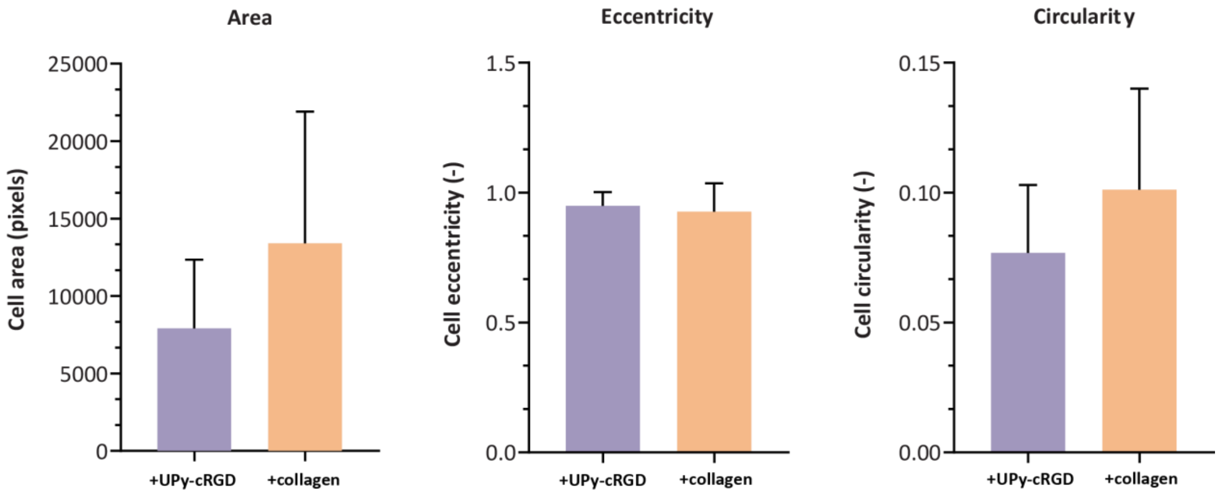

Figure S12. Encapsulated PKs treated towards CSKs sensed similar mechanotransduction when encapsulated in the synthetic or hybrid hydrogel and cultured for 17 days. The cells used to study the YAP ratio were also used for some additional analyses, n=3. A) Cell area of the cells in pixels. B) Cell eccentricity. C) Cell circularity.
